# Atypical presentation of anti-N-methyl-D-aspartate receptor encephalitis: two case reports

**DOI:** 10.1186/s13256-017-1388-y

**Published:** 2017-08-16

**Authors:** Maria Cristina Maggio, Greta Mastrangelo, Aldo Skabar, Alessandro Ventura, Marco Carrozzi, Giuseppe Santangelo, Francesca Vanadia, Giovanni Corsello, Rolando Cimaz

**Affiliations:** 1 Department of Rheumatology, Meyer Children’s Hospital, Florence, Italy; 2 Department of Advanced Diagnostic and Clinical Trials, Institute for Maternal and Child Health, IRCCS “Burlo Garofolo”, Trieste, Italy; 3 Department Pro.Sa:M.I. “G. D’Alessandro” ARNAS, University of Palermo, Palermo, Italy; 4 Unit of Paediatric Neuropsychiatry, Children Hospital “G. Di Cristina”, ARNAS, Palermo, Italy

**Keywords:** Anti-N-methyl-D-aspartate receptor encephalitis, Juvenile idiopathic arthritis, Teratoma, Psychiatric symptoms, Chorea, Speech disorders

## Abstract

**Background:**

Anti-N-methyl-D-aspartate receptor encephalitis is a rare autoimmune disease characterized by severe neurological and psychiatric symptoms and a difficult diagnosis.

The disease is often secondary to a neoplastic lesion, seldom diagnosed years later. Psychiatric symptoms are prevalent in adults; neurologic symptoms are more evident in children, who typically present primarily with neurological symptoms. To the best of our knowledge, the association with juvenile idiopathic arthritis has not been described.

**Case presentation:**

We report the cases of two caucasian girls with an atypical presentation.

The first patient was an 8-year-old girl with normal psychomotor development. Over a 4-month period she developed behavioral problems, speech impairment, and deterioration in academic skills. Within 8 months from the onset of symptoms, choreic movements gradually appeared. Hematological, neuroradiological, and neurophysiological examinations were negative; however, her symptoms worsened and treatment with prednisone was started. Although her choreic movements improved within 1 month, her neuropsychological and behavioral symptoms continued. Anti-N-methyl-D-aspartate receptor antibodies in cerebrospinal fluid and in blood were detected. Therapy with intravenously administered immunoglobulins was administered, without improvement of symptoms. After 2 months of steroid treatment, she suddenly started to pronounce some words with a progressive improvement in language and behavior.

The second patient was a 14-year-old girl with classic anti-N-methyl-D-aspartate receptor encephalitis, treated successfully with intravenously administered immunoglobulins and methylprednisolone, followed by orally administered prednisone, who developed chronic arthritis of the hip. The arthritis was confirmed by magnetic resonance imaging and associated to antinuclear antigen antibody positivity.

One year after the encephalitis presentation, an ovarian cystic mass was identified as a teratoma. The surgical resection of the mass was followed by the resolution of the psychotic spectrum and arthritis.

**Conclusions:**

Anti-N-methyl-D-aspartate receptor encephalitis in pediatric patients can present initially with neuropsychological and behavioral symptoms.

In the literature, the association of anti-N-methyl-D-aspartate receptor encephalitis with juvenile idiopathic arthritis is not yet described: to the best of our knowledge, this is the first case reported. The link to a neoplastic lesion can explain the favorable course of encephalitis and arthritis, after the surgical resection of the mass. Early diagnosis and treatment can improve the patient’s outcome.

## Background

Anti-N-methyl-D-aspartate receptor (NMDAR) encephalitis is a recently described disease characterized by five stages of development (prodromal phase, psychotic phase, unresponsive phase, hyperkinetic phase, and gradual recovery phase), with a prevalence of psychiatric symptoms in adult patients and neurological symptoms in pediatric patients.

The presenting features are: behavioral changes, psychosis, seizures, oro-lingual-facial dyskinesia, extreme irritability, and insomnia. The symptoms are persistent, and the course is usually progressive over 4 to 8 weeks’ duration [1–3].

The diagnosis is based on detection of anti-NMDAR antibodies, since neuroimaging and electroencephalography (EEG) are nonspecific.

An atypical presentation of the disease in children can result in a delay in diagnosis and appropriate treatment. Intravenous pulse methylprednisolone and immunoglobulins are the first-line treatment. Some children require second-line immunotherapy. In the literature rituximab is reported as useful, and the response to cyclophosphamide pulse therapy is satisfactory. Although the response to corticosteroids and/or immunoglobulins can be quite variable, early diagnosis of the illness can be crucial in improving the patient’s outcome. A tumor screen is mandatory in all children to detect neoplastic lesions often found to be etiologically linked to the encephalitis. Ovarian teratoma is often associated [4]. The outcome depends on the histological type, the spectrum of associated autoimmune diseases, and on prompt treatment with immunotherapy.

## Case presentation

### Case 1

The first patient was a healthy 8-year-old caucasian girl. Her parents were divorced and she lived with her mother, but she saw her father every week. At 7 years of age, she had developed cystitis with urinary incontinence. The condition resolved; however, her behavior changed when she started school. She became agitated and hyperactive and experienced frequent episodes of tachycardia. She would wake up in the middle of the night and jump on her bed. In addition, she became very anxious and attached to her mother, and she continuously asked her mother if she loved her. Moreover, she exhibited frequent bouts of laughing and crying during the day for no apparent reasons. She spoke “like a little girl” (baby talk) and her academic skills progressively worsened. A pediatric neurologist diagnosed her with anxiety. An EEG recording during wakefulness was normal, while neuropsychological testing showed a deficit in executive functioning. After a few months she developed symptoms of dysarthria and eventually stopped speaking altogether 2 months later. She subsequently developed a low-grade fever and a complex partial seizure. Subsequently hospitalized under our observation she presented as impatient and hyperactive. She was very communicative in non-verbal ways and excessively friendly, but anxious at the same time. She did not sleep, but when she did fall asleep, she would wake up frequently. Although she did not speak, the communicative intent was clear. Her gestures were used appropriately, but her only verbal utterance was to say in an explosive way “mama” and “no.” Her verbal comprehension was preserved even for the most complex requests. Choreiform dyskinesia was present in her facial muscles and limbs.

Diagnostic testing including magnetic resonance imaging (MRI) and computed tomography (CT) scan of her brain, blood levels of copper, throat swab, blood and urine levels of amino acids, electrocardiogram (EKG), echocardiography, and repeated EEG during sleep and wakefulness were all normal. Antistreptolysin O titer, complete blood count, liver and renal function tests, and inflammation and infection markers were in the normal range.

Symptomatic treatment with haloperidol (0.1 mg/kg) was started and her neurological symptoms quickly improved. After 2 days, her choreic movements were reduced as shown by Sydenham’s Chorea Rating Scale (USCRS), in which total score decreased from 4 to 2. Approximately 10 days after beginning treatment, her choreic movements decreased further. Language, however, was still limited to a few words. Sleep disturbances were still present even when her hyperactivity and disinhibition were reduced. Treatment with prednisone at a dose of 2 mg/kg per day was started. Her choreic movements disappeared completely and her parents reported that she was able to speak a few sentences when she sang. Treatment with haloperidol was then stopped and corticosteroid treatment continued. After approximately a month of therapy with prednisone the clinical picture was still characterized by a significant impairment in speech and the persistence of behavioral symptoms.

A lumbar puncture was then performed because we suspected a diagnosis of autoimmune encephalitis. A cerebrospinal fluid (CSF) examination showed oligoclonal bands with a type 3 pattern and the presence of anti-NMDAR antibodies. CSF glucose and proteins levels were within normal limits: 44 mg/dl (normal values 32 to 82 mg/dl) and 16.60 mg/dl (normal values 15 to 45 mg/dl), respectively. No cells were found. Anti-NMDAR antibodies were also present in a blood sample. Ultrasound excluded the presence of ovarian teratoma and a chest X-ray was normal. Therapy with intravenously administered immunoglobulin (IVIG) at the dose of 1 g/kg was given and corticosteroid treatment continued. After 2 months of the IVIG infusion, she started to pronounce words and within a few days, language was completely restored. Four weeks later, steroid therapy was reduced to 12.5 mg/day; however, she again began to have difficulties in speech production and the therapy was resumed. Neurological problems eventually disappeared. Nine months later, steroid therapy was reduced and finally discontinued. One year later, speech was restored and she was completely asymptomatic and no further relapses occurred.

### Case 2

A 14-year-old caucasian girl presented to our unit with an acute onset of neuropsychiatric disorders, characterized by a severely agitated state, headache, speech and swallowing difficulties, generalized seizures, which in a few days developed into a severely agitated catatonic state with opisthotonic posturing, tonic posturing of limbs, insomnia, and dyskinesia. She further manifested loss of sensitivity in the distal portion of her left leg.

She also developed left hip arthritis, with pain and functional impairment of articular movements. The arthritis was confirmed by MRI, showing joint effusion and synovial membrane hypertrophy. Brain MRI was free from abnormalities; CSF analysis was in the normal range for leukocytes, proteins, and anti-NMDAR antibodies; equally negative was CSF detection of herpes simplex virus type 1 (HSV-1) by polymerase chain reaction (PCR). EEG showed focal slow-waves in the front central area.

Serum antinuclear antigen antibodies (ANA) were positive, with a titer of 1:160; serum anti-NMDAR antibodies were also positive, while anti-cardiolipin antibodies and lupus anticoagulant were negative. Human leukocyte antigen (HLA)-B27 allele was absent. Alpha-fetoprotein, beta-human chorionic gonadotropin (beta-hCG), CA125, and CA19.9 were evaluated periodically during the follow-up and always showed concentrations in the physiological range.

An abdominal and pelvic scan, and chest, abdomen and pelvis CT were evaluated for occult malignancy. However, initially they did not reveal any neoplastic lesion.

She started a 3-day course of high-dose pulse intravenous methylprednisolone (30 mg/kg per day), followed by orally administered prednisone (2 mg/kg per day weaned over 3 months) and IVIGs 400 mg/kg per day for 5 days. Further, she received haloperidol for her movement disorders. Her psychiatric and neurological symptoms resolved; however, the loss of feeling in her leg was lasting.

She received non-steroidal anti-inflammatory drugs for the arthritis persistence, with a slow reduction of pain, functional limitation, and swelling.

Furthermore, a relapse of psychiatric symptoms occurred with agitated catatonic state and dyskinesia.

She was treated with a second course of steroids (orally administered prednisone 2 mg/kg per day) and IVIG (400 mg/kg per day for 5 days). Ultrasound, CT, and MRI screening for occult malignancy were repeated and revealed an ovarian teratoma. The teratoma was surgically removed, with a resolution of her psychiatric and neurological manifestations.

A second flare of arthritis occurred, and resolved after 1 month of treatment with non-steroidal anti-inflammatory drugs.

## Discussion

Anti-NMDAR encephalitis is a disorder caused by antibodies to the NR1 subunit of the NMDAR and has been recognized as the most frequent autoimmune encephalitis in children after acute demyelinating encephalomyelitis [5]. The encephalitis is associated with the production of auto-antibodies directed to NMDAR, a protein involved in memory function and synaptic plasticity. The patients’ antibodies have in fact pathogenic effects on the NMDAR.

Anti-NMDAR encephalitis is characterized by the presence of neurological and psychiatric symptoms. Isolated psychiatric symptoms are rare at the onset of the disease in children. They are more frequent in adults and more typical during relapses [1]. Patients develop neurological and/or psychiatric symptoms 2 to 4 weeks following the prodromal phase of the illness (usually upper respiratory tract symptoms, fever, nausea, headache, vomiting, or diarrhea) [2, 6]. The psychiatric symptoms in adults and in adolescents are usually characterized by anxiety, delusional thinking, mania, paranoia, and visual and auditory hallucinations. Sleep disorders, especially insomnia, are also common. In contrast, children present more frequently with irritability, bouts of inappropriate laughing or crying, hyperactivity, temper tantrums, and violent behaviors, in addition to insomnia and anxiety. Deterioration in speech is frequently reported in both in pediatric and adult ages as an initial symptom [1]. In the 12 years and under age group, the initial symptoms are usually neurological, consisting of dystonia, dyskinesia, and/or seizures (usually partial seizures) [2].

In the 32 patients described by Florance *et al*. [1], 84% and 77% showed movement disorders and seizures respectively, and 59% showed behavioral/personality disorders. In another series, 67% of patients younger than 12 years of age initially showed neurologic symptoms and 33% psychiatric symptoms [7].

The EEG either at the onset or during the disease course is abnormal in 90 to 100% of cases [8]. The sensitivity of MRI is lower, being positive in 30 to 55% of cases [6].

In their pediatric series of 20 cases, Armangue *et al*. [2] grouped the symptoms of anti-NMDAR encephalitis into nine categories: behavioral dysfunctions, sleep dysfunction, memory deficit, speech dysfunction, movement disorder, seizures, loss of consciousness, autonomic dysfunction, and central hypoventilation. They showed that within the first month of the disease each patient developed a median number of symptoms equal to 5.5 (range 4 to 8). Psychiatric dysfunction, impaired speech, and abnormal movements (usually orofacial dyskinesia and choreoathetosis) developed at varying timeframes over the course of the illness.

Patient 1 showed all the neurological, neuropsychological, and psychiatric symptoms typical of NMDAR encephalitis, although the timing of their appearance was atypical and neuroradiological and electroencephalographic examinations were negative. In fact, she was referred for our attention 9 months after the onset of psychiatric symptoms, and child abuse had been hypothesized as a possible cause for her clinical presentation. The sole initial neurological symptom in case 1 was a complex partial seizure, which occurred 8 months after the onset of disease. The dystonic/dyskinetic movement disorder became fully expressed after another month. EEG and MRI remained negative, even when neurological signs and symptoms were present.

The disease can be linked to neoplastic lesions, more frequently gonadic teratoma. These cases are considered a paraneoplastic syndrome, and surgery of the teratoma can resolve neurological symptoms. Surgery probably removes antigenic stimulus of autoimmunity. Especially in teratoma several different tissues are detected, with possible autoimmune cross-mimicry [9]. However, in many cases the tumors are not detectable and the patients have a clinical course linked to the pharmacological treatment.

The disease has a well-delineated set of clinical features, with neuropsychiatric expression. In the case 2, behavior changes were associated with agitation, headaches, memory deficit, and speech problems. She also showed bizarre movements of hands and legs and seizures.

As an autoimmune disease, an association with other autoimmune and/or rheumatologic diseases could be observed.

In the literature, only one case of anti-NMDAR encephalitis in an adult patient, with long-term seropositive rheumatoid arthritis (RA), was described. This case showed an atypical presentation which developed while she was under biologic therapy [10].

The association with juvenile idiopathic arthritis (JIA) has not, however, been described yet: to the best of our knowledge, this is the first case reported in the literature. Furthermore, the link to a neoplastic lesion can explain the favorable course after the surgical resection of the mass and the favorable course of arthritis as well.

## Conclusions

For the acute psychosis, many patients are first followed by psychiatrists: pediatricians and psychiatrists must consider anti-NMDAR encephalitis as a possible cause of acute psychosis in children and adolescents without a history of psychiatric events [5, 8].

In these patients, an accurate follow-up must exclude an occult neoplasm; the diagnostic pattern must include imaging with neoplastic biomarkers (such as CA125, CA19.9) for the identification of the most common neoplasm: ovarian teratoma [11]. However, a normal range of CA19.9 does not exclude a teratoma, as documented in our patient.

The association of other autoimmune diseases could help physicians with the diagnostic path.

Finally, although it is known that symptoms respond after a long period of time to immunomodulatory therapy with corticosteroids and immunoglobulins, it remains questionable if a timely treatment start could shorten the neuropsychological and neurological recovery.

In conclusion, in the absence of neurological symptoms and a negative EEG, anti-NMDAR encephalitis must be kept in mind as a differential diagnosis in a young child presenting mainly with psychiatric symptoms. Initiation of appropriate treatment in a timely manner may result in a most favorable outcome.

Furthermore, the possible coexistence of other autoimmune diseases in these patients stresses the usefulness of a long-term follow-up from the pediatric age to young adulthood for the precocious recognition of other associated autoimmune diseases.

## Abbreviations

ANA: Antinuclear antigen antibodies
beta-HCG: Beta-human chorionic gonadotropin
CSF: Cerebrospinal fluid
CT: Computed tomography
EEG: Electroencephalography
EKG: Electrocardiogram
HLA: Human leukocyte antigen
HSV-1: Herpes simplex virus type 1
IVIG: Intravenously administered immunoglobulin
JIA: Juvenile idiopathic arthritis
MRI: Magnetic resonance imaging
NMDAR: N-methyl-D-aspartate receptor
PCR: Polymerase chain reaction
RA: Rheumatoid arthritis
USCRS: Sydenham’s Chorea Rating Scale
